# Shifting the phase of a coherent beam with a $^{174}\hbox {Yb}^+$ ion: influence of the scattering cross section

**DOI:** 10.1007/s00340-016-6609-3

**Published:** 2017-01-16

**Authors:** Martin Fischer, Bharath Srivathsan, Lucas Alber, Markus Weber, Markus Sondermann, Gerd Leuchs

**Affiliations:** 1 Max-Planck-Institute for the Science of Light, Staudtstr. 2, 91058 Erlangen, Germany; 2 Department of Physics, Friedrich-Alexander University Erlangen-Nürnberg (FAU), Staudtstr. 7/B2, 91058 Erlangen, Germany; 3 Department of Physics, University of Ottawa, 25 Templeton, Ottawa, ON K1N 6N5 Canada

**Keywords:** Phase Shift, Scatter Cross Section, Scattered Field, Incident Field, Evanescent Field

## Abstract

We discuss and measure the phase shift imposed onto a radially polarized light beam when focusing it onto an $^{174}\text {Yb}^{+}$ ion. In the derivation of the expected phase shifts, we include the properties of the involved atomic levels. Furthermore, we emphasize the importance of the scattering cross section and its relation to the efficiency for coupling the focused light to an atom. The phase shifts found in the experiment are compatible with the expected ones when accounting for known deficiencies of the focusing optics and the motion of the trapped ion at the Doppler limit of laser cooling (Hänsch and Schawlow in Opt Commun 13:68–69, 1975).

## Recollection by one of us (GL)

Birthdays often evoke memories of the one who is celebrating. Sometimes it is a single question they have asked you that has stuck in your mind for a long time. Of the many times I met Ted Hänsch one comes to my mind in particular. It was when I saw him in a corridor at the Max Planck Institute of Quantum Optics, about thirty years ago—the building was quite new at the time. I vividly remember the question he asked me: ‘Do you have a good explanation why the cross section of an atom for scattering light is as large as it is?’ He was referring to the classical on-resonance cross section of an atom, $\sigma _{\text {sc}}=3\lambda ^2/2\pi$, being so much larger—i.e. many orders of magnitude—than the cross section of the atomic charge distribution. Naturally, I knew the phenomenon and answered that in scattering processes the larger of the two following values dominates: the cross section of the atom as a massive object or the cross section of the particle you send in to probe the atom, namely a photon in the case under consideration. Obviously, the smallest cross section of an optical beam is limited by diffraction and this, I had thought, should define the cross section of the photon. I was surprised to see that Ted Hänsch did not seem satisfied as he slowly turned away. At the time, this made me think, and throughout the years since then I have returned to this thought every now and then.

Ten years later, after I moved to Erlangen, this ‘thinking’ became more intense when within my group we started to first discuss spontaneous emission and the possibility of observing its time-reversed counterpart. In spontaneous emission, the energy is initially concentrated in a tiny volume, which is orders of magnitude smaller than the wavelength cubed—partially still stored in the atom—and begins to travel outwards. At first, the energy is both in the evanescent and propagating components of the field. Then, as the outgoing dipole wave travels further, the evanescent components decay away leaving only the propagating part of the dipole wave. The idea arose that the evanescent field is more part of the atom than of the outgoing dipole wave. Consulting any book on electromagnetism, one can calculate the outward going energy flux of the near field and that of the propagating field. The near-field part quickly decreases to zero as the distance to the source increases, whereas the far-field component is constant. It is interesting to note that the radial position *r*, at which the energy flux of the near field has reduced to half the far-field portion, is given by $(2\pi /\lambda )^2r^2\sim 6$. This *r* value corresponds exactly to the above-mentioned cross section, indicating that in terms of cross section the near field can be considered part of the atom. Was this good enough to tell Ted Hänsch? Without the atom, light would produce a diffraction-limited spot, but when an atom is at the origin of the dipole wave, one instead expects time-reversed spontaneous emission to occur such that the energy density of the field increases far beyond the diffraction-limited value in free space. One might speculate that the evanescent field is excited via the atom’s reaction to the incident field. If one considers the evanescent field as part of the atom, its extent defines the cross section of the atom, resulting in a cross section almost matching the classical textbook value quoted above. Nevertheless, at that point I still felt it was too early to go back to Ted Hänsch. There was still something that puzzled me.

The incoming dipole wave with its evanescent and propagating components is an exact solution of Maxwell’s equations, but it has a singularity. Accordingly, when one excites an inward propagating dipole wave in the far field, one would expect the singularity to develop—this is part of the rigorous solution after all—up to the point when the wave reaches the atomic charge distribution. We know however that this is not what happens. Thus, it was a great relief to me when Simon Heugel, a doctoral student in our group at the time, came to me about seven years ago suggesting that I look at problem C$_I$.6 in the text book by Cohen-Tannoudji, Dupont-Roc and Grynberg [2]. There it is stated that in free space the inward propagating dipole will continue as an outward propagating dipole once it has passed the origin and will thus interfere with itself. The task given to the students is to calculate the energy density of the resulting standing wave and—alas—the result is the diffraction-limited field distribution, provided one takes into account a phase shift at the origin which is in a way the Gouy phase shift under this extreme full solid angle focusing scenario. Looking at the problem in this way everything seems to fall into place: (1) when focusing in free space, the singular terms in the dipole wave solution interfere destructively and (2) suppressing the outward going wave via full absorption at the origin by a sub-wavelength antenna such as an atom gives rise to the well-known field enhancement. We asked ourselves whether there are other ways to restore the singular behavior. One way we found theoretically was by studying the time evolution of the energy distribution for focusing in free space near the origin when the inward going dipole wave has a sharp rising leading edge, rising over a distance significantly smaller than the wavelength. This indeed also gave a transient enhancement [3]. Other experiments are under way.

Encouraged by these considerations and findings we hope this anniversary is the right moment to give Ted Hänsch an update on our, by now decades long, attempt to answer the question he posed such a long time ago.

## Introduction

The *scattering cross section* is a quantity used in many areas in physics, relating the rate of particles scattered by a target to the flux of particles incident onto it. In quantum optics, the conceptually simplest target is a single atom and the incident particles are photons. For this scenario, the resonant scattering cross section for a two-level atom is determined to be [4, 5]

1 $$\begin{aligned} \sigma _{\text {sc}} = \frac{3}{2\pi }\lambda ^2 \end{aligned}$$

for an atomic transition with resonance wavelength $\lambda$ provided the oscillator strength [6] is equal to one.

As mentioned above, the area given by $\sigma _{\text {sc}}$ is large: It is by far larger than the spatial extent of an atom given by the Bohr radius and also larger than the smallest spot sizes achievable via diffraction-limited focusing of light with lenses of sufficient numerical aperture [7, 8]. The term cross section was created to describe scattering of particles, but in wave mechanics there is also the interference of fields. As pointed out in Ref. [9], absorption can be described as the interference of the (non-attenuated) incident field and the scattered field. In this model, attenuation in forward direction is caused by the destructive interference between these two fields, which requires a power of the scattered field which may seem counter-intuitive at first sight: full attenuation, and only back-scattered light requires the power of the scattered field to be twice that of the incident field because of the destructive interference with the incident light in the forward direction, in order to fulfill energy conservation. Along those lines, the rate of scattered photons, which is not to be confused with the detected photons, expressed in terms of cross sections is given by [10]

2 $$\begin{aligned} \gamma _{\text {sc}}=\frac{\sigma _{\text {sc}}}{A}\cdot \gamma _{\text {inc}}, \end{aligned}$$

with *A* denoting the effective mode area [10, 11] of the incident stream of photons $\gamma _{\text {inc}}$. The remarkable scenario of more photons being scattered than photons arriving, both per unit time [12], arises when $\sigma _{\text {sc}}$ becomes larger than *A*. Due to the interference of the different outward going partial waves, energy conservation is, of course, maintained. Within this reasoning, several intriguing phenomena occurring in the interaction of light and single quantum emitters have been investigated in recent years; see Ref. [13] for a review. However, as reported in Ref. [14] it was found already in the early 1980s by Bohren [15] and Paul and Fischer [16] that an atom can scatter more light than incident onto its massive cross section, which is on the order of the Bohr radius squared. As also discussed in more recent publications, the key step in these papers was indeed the examination of the superposition of incident and scattered fields. Refs. [15, 16] revealed that within a certain area larger than the size of the scatterer the resulting lines of energy flux end up at the scatterer’s position. Within a similar reasoning and as outlined in the first section of this paper, one could attribute the spatial extent of the non-propagating near-field components of the field re-radiated by the atom to the size of the atom, leading to the expression for $\sigma _{\text {sc}}$ given by Eq. 1.

Here, we relate to such concepts by investigating the phase shift imprinted onto a tightly focused light beam by a single $^{174}\text {Yb}^{+}$ ion. In the next section, the importance of the magnitude of the effective mode area of the incident beam to the obtained phase shift is revisited. With simple arguments, we modify the equation obtained in Refs. [13, 17] describing the achievable phase shift to account for the level structure of the used ion species. Explicitly, we make use of the dependence of the scattering cross section on the angular momenta of the involved atomic levels. In Sect. 4, we describe our experimental apparatus, present the phase shift observed in our experiments and compare the obtained results to the predictions of Sect. 3. At the end of the paper, we give concluding remarks.

## Relation of scattering cross section and phase shift

In order to emphasize the role of the scattering cross section $\sigma _{\text {sc}}$ in phase shifting a weak coherent beam, we briefly recall some essential aspects. Typically, the induced phase shift is considered as the phase difference of the superposition of the incident electric field $E_{\text {inc}}$ and the scattered field $E_{\text {sc}}$ relative to the phase of the incident one, i.e. the phase of the incident field leaving the interaction region when no atom is present [18–20]. The phase shift $\Delta \varphi$ can then be written as [18]

3 $$\begin{aligned} \Delta \varphi =\arg \left( \frac{E_{\text {inc}}+E_{\text {sc}}}{E_{\text {inc}}}\right) \end{aligned}$$

with $\arg (\,)$ denoting the argument of its complex variable.

Since one is considering a coherent process in this situation, it is detrimental to saturate the atomic transition, i.e. to produce incoherent components in the scattered radiation. We therefore assume negligible saturation. For this case, the phase shift imprinted by a *two-level* atom is found to be [17]

4 $$\begin{aligned} \Delta \varphi =\arg \left( 1-2G\cdot \frac{1+i\cdot 2\Delta /\varGamma }{1+4\Delta ^2/\varGamma ^2}\right) \, . \end{aligned}$$

Here, $\varGamma$ denotes the spontaneous emission rate and $\Delta$ is the detuning between the laser and the atomic resonance frequency. At fixed detuning, the crucial parameter determining the magnitude of the imprinted phase shift is *G*, describing the extent to which the atom experiences the highest possible electric field at constant input power which is allowed for by diffraction: $G= E_{\text {inc}}^2/E_{\text {max}}^2$, where $0\le G\le 1$. $E_{\text {max}}$ is the field amplitude obtained by focusing a dipole wave in free space [21], i.e. *G* determines how efficiently the incident field couples to the atomic dipole transition. Assuming an atom at rest, *G* is solely determined by the properties of the focusing optics and the spatial mode of the incident field which has an overlap of $\eta$ with the field of the driven transition [13, 22], $G\propto \eta ^2$. It also accounts for phase front aberrations that are induced by imperfect focusing optics [23, 24]. Therefore, *G* is a measure for the quality of the mode matching of the incident mode to the atomic dipole-radiation pattern.

The role of *G* becomes obvious when relating it to the so-called *scattering ratio* on resonance, which is defined as $R=\gamma _{\text {sc}}/\gamma _{\text {inc}}$ [10, 18]. One can show that in general $G=R/4$ [13], resulting in

5 $$\begin{aligned} G=\frac{\sigma _{\text {sc}}}{4A} \, . \end{aligned}$$

Hence, in order to reach unit coupling efficiency and thus the maximum phase shift at a fixed, nonzero detuning, the effective mode area of the focused beam must not be larger than a quarter of the scattering cross section. One can actually show that $\sigma _{\text {sc}}/4$ is the minimum possible mode area in free space. We interpret the effective mode area $A$ as the power $P$ of the incident light divided by the intensity $I$ at the position of the atom. With the help of Eqs. 11 and 12 from Ref. [13] one can directly obtain $A=3\lambda ^2/(8\pi G)$ and thus $A=\sigma _{\text{sc}}/(4G)$, which is minimized in free space by $G=1$.

Inserting Eq. 5 into 4 results in

6 $$\begin{aligned} \Delta \varphi =\arg \left( 1-\frac{\sigma _{\text {sc}}}{2A} \cdot \frac{1+i\cdot 2\Delta /\varGamma }{1+4\Delta ^2/\varGamma ^2}\right) \, , \end{aligned}$$

similar to the findings of Ref. [18]. On resonance, the phase of the outgoing field can only take two values: zero if $A\ge \sigma _{\text {sc}}/2$ and $\pi$ as soon as the electric field is focused to a spot smaller than $\sigma _{\text {sc}}/2$. This representation reveals that the obtainable phase shift is not only limited by imperfect focusing, as expressed by a too large *A*. But also choosing the ‘ideal’ atom is of importance, i.e. an atom for which Eq. 1 is valid. Deviations could originate from a degenerate ground state as is the case for $^{174}\text {Yb}^+$ or from an atom not being at rest [5]. Both obstacles occur in the experiment presented in the next section.

**Fig. 1:**
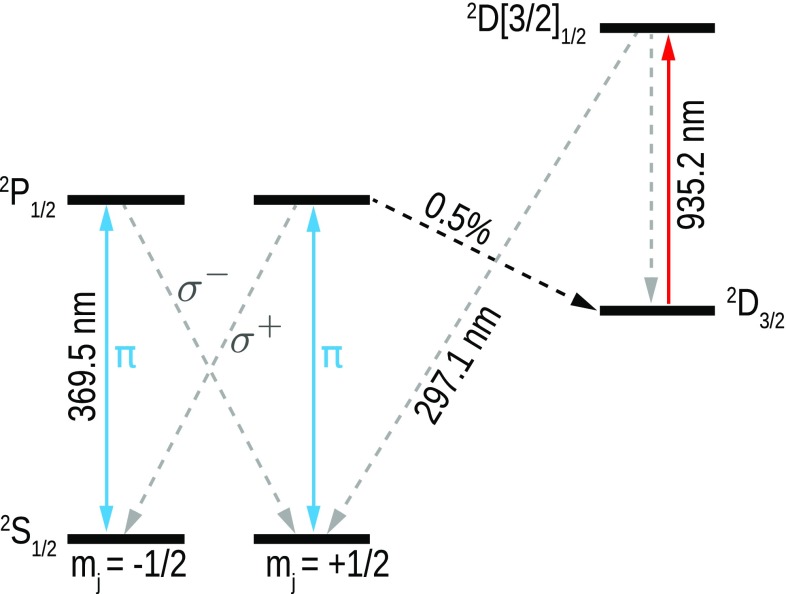
Level scheme of $^{174}\text {Yb}^{+}$. In the phase-shift experiments, we drive the $\pi$-transition between the $S_{\text {1/2}}$ and the $P_{\text {1/2}}$ state. Furthermore, we use optical pumping to prepare the ion in the metastable $D_{\text {3/2}}$ (dark) state for obtaining a reference phase. The branching ratio from the $P_{\text {1/2}}$ state into the $D_{\text {3/2}}$ state is $0.5\,\%$ [25]

In the remainder of this section, we explicitly treat the level structure. In general, when accounting for the sub-structure of the atomic levels involved, the resonant scattering cross section of an atomic transition can be written as [5]

7 $$\begin{aligned} \sigma _{\text {sc}}=\frac{\lambda ^2}{2\pi }\cdot \frac{2J'+1}{2J+1} \end{aligned}$$

with $J'$ and *J* being the total angular momentum of upper and lower level, respectively. For our experiment involving the $\text {P}_{1/2}\leftrightarrow \text {S}_{1/2}$ transition of $^{174}\text {Yb}^+$ (cf. Fig. 1), we have $J'=J$ and hence $\sigma _{\text {sc}}=\lambda ^2/(2\pi )$, which is only 1 / 3 of the value used so far. We explicitly account for this reduction of the scattering cross section in writing

8 $$\begin{aligned} \Delta \varphi _{J=J'}=\arg \left( 1-\frac{2G}{3}\cdot \frac{1+i\cdot 2\Delta /\varGamma }{1+4\Delta ^2/\varGamma ^2}\right) \, . \end{aligned}$$

Consequently, *G* from now on only accounts for imperfect focusing and atomic motion.

The result of Eq. 8 can also be obtained from a solution of the Bloch equations for a $J'=1/2\leftrightarrow J=1/2$ system driven only by a $\pi$-polarized light field. The modification $G\rightarrow G/3$ can be understood as follows. First, the dipole moment in excitation is reduced by a factor $1/\sqrt{3}$ in comparison with a two-level atom. Second, the amplitude of the coherently scattered field that can interfere with the incident radiation is reduced by the same factor, because the $\sigma ^\pm$-components of the scattered field cannot interfere with the incident light. A detailed calculation will be presented somewhere else.

## Setup and experiment

**Fig. 2:**
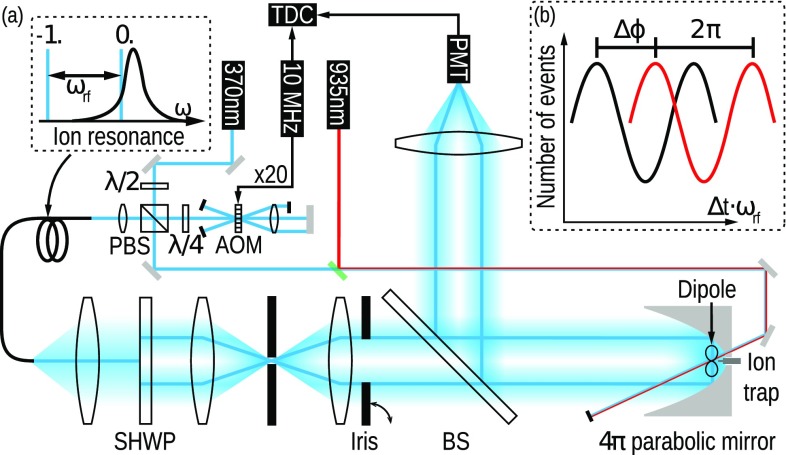
**a** Setup for measuring the phase shift imprinted by a single $^{174}\text {Yb}^+$-ion. With an additional laser beam at a wavelength of 935.2 nm, we can pump the ion back from the metastable $D_{\text {3/2}}$ state into the $S_{\text {1/2}}$ ground state. This laser is sent onto the ion from a hole at the backside of the parabolic mirror. The same is done for a second laser beam at a wavelength of 369.5 nm that is used for ionization and for cooling the ion in certain steps of the experimental procedure (see text). *SHWP* segmented half-wave-plate, *(P)BS* (polarizing) beam splitter (other abbreviations in the text). **b** Intensity signal $I_{\text {TDC}}(t)$ obtained from the statistics of photon detection times on the TDC for the ion being in the bright state (*red*) and in the $D_{\text {3/2}}$ dark state (*black*)

In our setup, we utilize a parabolic mirror as the focusing device [23, 24, 26]. The parabolic mirror tightly focuses a radially polarized donut mode to a field that is linearly polarized along the optical axis [7, 27]. This field drives a linear dipole oriented in the same direction.

We position the $^{174}\text {Yb}^+$ ion in the focus of the mirror by means of a movable open-access ion trap [26]. The focused donut mode continuously drives the linear dipole of the $S_{\text{1 }/2}$ to $P_{\text{1 }/2}$ transition of the ion, with a linewidth of $\varGamma /2\pi =19.6\,\text {MHz}$ [25], at a wavelength of 369.5 nm. The power of this beam is chosen such that saturation effects are negligible. In Ref. [24], it was found that the aberrations of the parabolic mirror used are so strong in the outer parts that it is favorable to focus only from the ‘backward’ half space when not correcting for these aberrations. We therefore decided to use this focusing configuration in the experiment reported here, by inserting a suitable iris in the excitation beam path, cf. Fig. 2. The iris has a radius of two times the focal length of the paraboloid. We refer to this configuration as focusing from half solid angle, since the bore in the vertex of the parabolic mirror, housing the trap, reduces the solid angle, relevant for the linear dipole, by less than 0.5%. The focused donut mode also provides Doppler cooling for the ion. Auxiliary beams needed, e.g. for the initial ionization and trapping as well as the repumping beam (cf. Figs. 1,  2), are entering the focal region of the mirror through a small auxiliary hole close to the vertex of the parabola.

Each phase-shift measurement is preceded by the following sequence: First the ion is Doppler-cooled by an auxiliary beam red detuned by half a linewidth from the $\text {S}_{1/2}\leftrightarrow \text {P}_{1/2}$-transition. Then, this auxiliary beam is switched off and the donut mode drives the ion at half linewidth detuning. The ion is scanned through the focal region while monitoring the count rate of photons at 297 nm; see Fig. 1. The ion is positioned such that this count rate is maximized. Afterward, the auxiliary beam at 369.5 nm is switched on again for Doppler cooling. Switching this beam off again and setting the donut beam to the desired detuning, the phase-shift measurement is initiated.

In this measurement interval, the temperature of the ion is governed by the interaction with the donut beam. Hence, the temperature is explicitly depending on the detuning of the donut beam, as also discussed later. For a detuning of $\Delta =-\varGamma /2$, Doppler cooling theory [28] predicts a minimal temperature of the ion of about $T=\hbar \varGamma /2k_{\text {B}}=470\,\mu \text {K}$, where $\hbar$ is Planck’s constant and $k_{\text {B}}$ the Boltzmann constant. From experimentally measured point spread functions (see Ref. [24]) and the characteristics of our ion trap (trap frequencies of 480 and 1025 kHz in radial and axial direction, respectively), we determine an upper bound of the ion’s temperature to be 50% above the Doppler limit at half linewidth detuning.

The phase shift imprinted by the ion is measured in a common path interferometer by heterodyne detection. We illuminate the ion with the near-resonant carrier donut mode and an off-resonant sideband donut, similar to the technique applied in Ref. [19]. The sideband donut is red-detuned from the $\text {S}_{1/2}\leftrightarrow \text {P}_{1/2}$-transition by $\omega _{\text {rf}}/2\pi =400$ MHz (amounting to about 20 linewidths) by using the diffraction order ‘$-1$’ of an acousto-optical modulator (AOM) in double-pass configuration ($\omega _{\text {rf}} = 2\, \omega _{\text {AOM}}$, see Fig. 2). Except for the frequency difference, both beams have exactly the same properties and are in the same spatial mode that is focused onto the ion.

After interaction with the trapped ion, the beams are retro-reflected and recollimated by the parabolic mirror. We measure the beating signal of the two beams with a correlation measurement (Fig. 2) involving a photomultiplier tube (PMT), a time to digital converter (TDC), and a 10 MHz trigger signal that is synchronized to the AOM, respectively. The intensity signal $I_{\text {TDC}}(\Delta t)$ obtained from the statistics of photon detection times on the TDC is fitted with a function proportional to $\cos (\omega _{\text {rf}}\, \Delta t + \phi _1)$ with phase offset $\phi _1$. To infer the relative phase shift $\Delta \varphi$ of the near-resonant beam, we repeat the measurement and fitting procedure after preparing the ion in the metastable $\text {D}_{3/2}$ (dark) state by optical pumping (see Fig. 1). This second measurement delivers the reference phase offset $\phi _2$. The phase shift $\Delta \varphi$ of the near-resonant beam is finally calculated via $\Delta \varphi =\phi _1-\phi _2$. The acquisition of sufficient statistics for each data point takes approximately ten seconds.

**Fig. 3:**
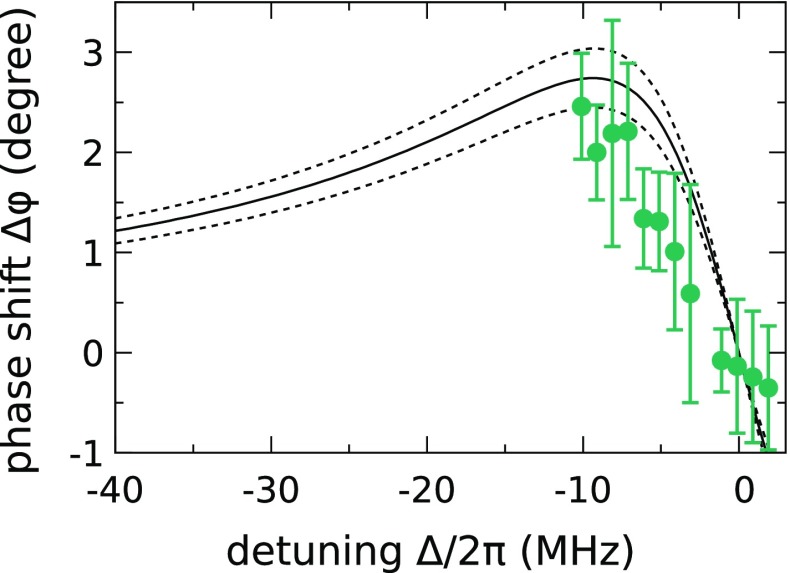
Measured phase shift $\Delta \varphi$ for different detunings (*symbols*) and phase shift according to Eq. 8 for a coupling efficiency of $G=13.7 \pm 1.4\,\%$ (*solid and dashed lines*). The value used for *G* is the one found in a saturation measurement in Ref. [24]

The results for measuring the phase shift as a function of detuning are shown in Fig. 3. We achieve a phase shift of $2.2^{\circ } \pm 0.5^{\circ }$ at approximately half linewidth detuning. These values are compared to the theoretically predicted values of Eq. 8 expected for a coupling efficiency of $G = 13.7 \pm 1.4\,\%$, found in an independent experiment based on a saturation measurement [24]. For detunings $-\varGamma /2\le \Delta \le 0$ the measured phase shift shows a systematic deviation from the theoretical model, which assumes a detuning independent coupling parameter *G*. There are three possible reasons for these deviations. Firstly, saturation effects are neglected for the theoretical curve based on Eq. 8. However, since the saturation of the transition was kept low during the measurements ($S<0.1$), the reduction of the measured phase due to saturation effects is expected to be less than 2%. Secondly, the observed phase shift drops faster than expected when going closer to resonance. A possible reason for this is that the temperature of the atom diverges when the detuning approaches zero [29] and consequently the size of the ion’s wave function increases [30]. This leads to a stronger averaging of the experienced electric field by the extent of the ions wave function [31] entailing a reduction of the coupling efficiency and therefore also of the measured phase shift. Lastly, measuring the phase shift via heterodyne detection leads not only to a phase shift of the close to resonant part of the two light fields focused on the ion but also to a nonzero phase shift of the $400\,\text {MHz}$ detuned sideband, acting as a phase reference. At about $400\;\text {MHz}$ detuning, this phase shift of the reference beam can be assumed constant over the measured data points and takes a value of approximately $0.13^\circ$ at a coupling efficiency of $G=13.7\%$, leading to an effective offset of the zero phase value, which is neglected in Fig. 3.

## Concluding remarks

The phase shift obtained in our experiments is among the largest phase shifts measured for a coherent beam interacting with a single emitter in free space so far [18–20]. Nevertheless, it still is far below the maximum possible value $\Delta \varphi =\pi$ which can be obtained on resonance for $G>0.5$ [13, 17, 32]. The lower phase shift demonstrated in our experiments is in parts due to the motion of the ion in the trap and the aberrations imprinted by the parabolic mirror, which made it necessary to focus only from half solid angle. The latter restriction limits the coupling to $G\le 0.5$ [13, 22]. But the more severe limitation is the choice of our atomic species with its reduced scattering cross section. Even for optimum focusing and cooling the ion to its motional ground state the imprinted phase shift will never be larger than 30$^\circ$— what still appears to be a fairly large value. Therefore, besides compensating mirror aberrations we aim at repeating our experiments with $^{174}\text {Yb}^{2+}$ [33], which offers the desired $J'=1\leftrightarrow J=0$ transition that enables the maximum scattering cross section.
